# Social freezing of oocytes: a means to take control of your fertility

**DOI:** 10.1080/03009734.2019.1707332

**Published:** 2020-01-14

**Authors:** Anna-Lena Wennberg

**Affiliations:** Nordic IVF, Gothenburg, Sweden

**Keywords:** Age, elective, fertility, oocyte, pregnancy, results, social egg freezing, vitrification

## Abstract

In the last few decades, there have been tremendous developments of assisted reproductive technologies, but the outcome of *in vitro* fertilization is highly dependent on the age of the oocyte. The introduction of vitrification offers a possibility to freeze eggs proactively in younger years and use them at later ages, also called social egg freezing. Knowledge about age-related fertility decline is insufficient among many women, and there is an overoptimistic trust that *in vitro* fertilization can overcome this. The awareness of proactive egg freezing is limited, both among doctors and in the general population. This review aims at increasing the knowledge about proactive egg freezing and offers a means to advise women correctly. It deals with national guidelines, the best age and optimal number of eggs to freeze, and the chance to succeed. Creating more public awareness about age-linked fertility decline and elective egg freezing may help women reproduce at their own pace – to take control of their fertility.

## Introduction

Ever since the birth of the first child from *in vitro* fertilization (IVF) in 1978 (1) there have been great developments in the area of assisted reproductive technology (ART). Many different ways to achieve parenthood exist, and there are now available treatments for most types of male and female infertility. However, a growing problem in the developed world is that we start families at a continuously later stage in life. The mean age for giving birth to the first child has increased all over the world. In Sweden it now reaches 29.4 years (2), with similar figures in all the Nordic countries. Parallel to this, female fertility drops with age. A recent study from Denmark showed that fertility decreases approximately 15% between 35 and 40 years and 35% annually above 40 years (3). Studies have shown that there is insufficient knowledge about age-related fertility decline (4–6). Nonetheless, many women are aware of their declining fertility potential, and it causes stress to women who have not yet found a partner to form a family with. On the other hand, the awareness of proactive egg freezing is limited, and there is often an overoptimistic belief that IVF will help to overcome age-related infertility (4,7). Creating more public awareness about age-linked fertility decline and elective egg freezing, also called social freezing, is therefore a key factor to help women reproduce at their own pace. Elective egg freezing can also serve as a means not only to have one child, but to increase the possibility of having a second and third child, for couples who have their first child fairly late.

### Egg freezing

Cryopreservation of spermatozoa, to protect male fertility, was established already in the 1950s (8), and techniques to cryopreserve embryos were developed during the 1980s (9). Because of their size, unfertilized eggs are more sensitive to cryopreservation than sperm and embryos. The first birth after oocyte freezing, followed by warming and IVF, was described already in 1986 by Chen in South Korea (10), but the results were difficult to reproduce. Cryopreservation techniques at that time were limited, with low oocyte survival after thawing, low fertilization rates, and poor pregnancy results. In later years a novel technique – vitrification – has been introduced, showing good warming results and with good fertilization and pregnancy rates (11). The technique means that the oocytes are cooled ultra-rapidly in liquid nitrogen to −196 °C, in a minimal volume of cryoprotectant. In addition, a hands-on tool has made the method easier to introduce into clinical practice than previously. Spanish and Italian groups have, in large randomized and cohort studies, demonstrated that live birth rates after IVF with vitrified oocytes are comparable to IVF using fresh oocytes (12,13). Importantly, international follow-up of children born after oocyte vitrification have not revealed any increased risk of adverse obstetric outcome or increased risk of birth defects (14,15).

Becoming pregnant with cryopreserved oocytes requires IVF. The woman’s ovaries need to be stimulated before the eggs can be collected and cryopreserved. After warming, the eggs are fertilized, with the partner’s or donated sperm, and an embryo is transferred to the uterus.

Oocyte freezing is valuable for a variety of medical reasons such as egg donation, genetic predisposition of premature ovarian failure, severe endometriosis, or any gonadotoxic treatment that may result in premature ovarian failure, e.g. cancer treatment. There may also be ethical or religious reasons to freeze unfertilized eggs instead of embryos. Elective egg freezing without medical reasons is somewhat more controversial, and there was a big debate when the method was introduced. However, most people today have a positive attitude towards the procedure. A Swedish questionnaire study among 2000 women of reproductive age showed that a majority were positive to elective egg freezing, both for medical and for non-medical purposes, and almost half of the women would consider the procedure themselves (16). The Swedish National Council of Medical Ethics (SMER) has also made a statement in favour of non-medical egg freezing (17), as have the European Society of Human Reproduction and Endocrinology (ESHRE) (18) and the Nordic Fertility Society (NFS).

Elective egg freezing for non-medical reasons was introduced in Sweden by Nordic IVF Gothenburg in 2011 and is now offered by most private clinics in Sweden. There is no specific law regulating oocyte cryopreservation in Sweden. National guidelines have therefore been created by the Swedish Society of Obstetrics and Gynaecology (SFOG) (19) based on guidelines from SMER (17) and ESHRE (18). The guidelines state that elective egg freezing for non-medical reasons is ethically acceptable provided that the woman receives thorough information about the procedure, the risks, and the chance of having a child by use of the cryopreserved eggs. The importance of information is also stressed in the recommendations by ESHRE, but the organization at the same time points out that it should be each woman’s free choice weather to freeze eggs or not.

According to SMER the cost for non-medical egg freezing should be covered by the individual. From 1 October 2013 drugs are not subsidized by the Swedish state for women who freeze eggs just by choice. One argument is that only a small percentage of the banked oocytes will probably ever be used (20–22), and the procedure is thus not cost-effective (23). It is, however, not obvious that this approach is equal and just. One can argue for various systems of public funding, part-funding, or pay-back systems for women who proactively freeze their eggs in younger years, similarly to funding IVF with public means (24). Younger women have better egg quality, better chance of becoming pregnant, and require a lower hormonal dose at stimulation than older women. It is also likely that is more cost-effective to try and get pregnant with own frozen young eggs than by egg donation at a more advanced age. Moreover, there are conditions that are neither purely medical nor non-medical, such as congenital low ovarian reserve or reduced ovarian reserve due to endometriosis, benign ovarian cysts, or unilateral adnexal operations. Egg freezing in this intermediate group is today not subsidized by the state.

### When to freeze?

The debate is ongoing regarding the suitable age for elective oocyte freezing. The biologically optimal time would be in the mid-20s, when fertility is at its peak, but it may not be cost-effective in relation to the low usage. In one study from the United States, a decision-tree model was constructed to determine the cost-effectiveness of oocyte preservation in comparison to no action at ages 25–40 years (25). In this model, a mathematical calculation was made of the probability of a live birth seven years after the decision of whether to freeze eggs or to take no action. The highest probability of live birth was seen when oocyte cryopreservation was performed at ages >34 years. Elective oocyte freezing compared to no action gave the greatest improvement in likelihood of live births at the age of 37 years (52% after oocyte freezing and 22% at no action), but there was little benefit over no action at ages 25–30 years. Furthermore, in this US setting, the cost-effectiveness for elective oocyte freezing was highest at the age of 37 years.

There is a distinction between effectiveness and cost-effectiveness, and it may be misleading to suggest an optimal age of 37 years for freezing in general. Advice regarding elective egg freezing should always be based on individual assessments. Also, based on data that follow, there may be reasons for women to consider egg freezing if they have not yet found a partner or are not planning a pregnancy when approaching the age of 35 years.

### How many eggs to freeze?

Another important issue is how many eggs to preserve in order to have at least one child. It is challenging to determine the ideal number of oocytes since it depends on many factors, including maternal age and health, ovarian reserve, reproductive goals, and also paternal health. No number of oocytes can offer a guarantee. Many clinics recommend banking around 20 eggs, which for most women means going through the procedure more than once. Two American prediction models, based on IVF/ICSI-treatments, estimate that approximately 20 oocytes are needed, to have around 75% likelihood of achieving at least one child provided that the woman is younger than 38 years (Table 1) (26,27). The models also show that the chance of success is highly age-dependent and confirm that attempting fertility preservation in women at an age of over 40 years is unlikely to succeed.

**Table 1.** Live birth predictions by age, with 20 mature oocytes banked (24).

Age (y)	Probability of one live birth (%)	Probability of two live births (%)	Probability of three live births (%)
34	90	66	38
37	75	39	15
42	37	7	1

Appropriate information about the potential to bank a sufficient number of oocytes is vital for women’s decision-making. Cryopreservation of too few oocytes would limit the possibility of success, while an excessive number would increase the physical discomfort and the expense. Most clinics use a combination of age, antral follicle count, and measurement of anti-Müllerian hormone (AMH) to advise women properly. Observing a low ovarian reserve at a young age would cause a stronger incitement to preserve oocytes, but it would also increase the cost because of the necessity to repeat the freezing procedure.

### What is the chance of having a child?

An appropriate question is evidently what the chance to have a child is. Previous studies have shown that frozen eggs are as good as fresh ones (12,13), but direct pregnancy results from non-medical elective egg freezing are scarce. To a great extent we have had to rely on outcomes from conventional IVF, but data from egg freezing are now coming up.

Cobo et al. (20) presented a series of 137 out of 1468 women who returned for autologous IVF. The women preserved eggs for both medical and non-medical reasons and constituted 9% of the total number of women freezing eggs electively. The cumulative ongoing pregnancy rate/live birth rate (OPR/LBR) per patient was almost 60%.

In an Australian study, 95 women who had stored oocytes for non-medical reasons answered a non-anonymous questionnaire (21). Six of these women had returned for autologous IVF (in total 6% of the responders), and three of them had given birth as a result.

At Nordic IVF Gothenburg we recently published our own data from 1 August 2011 to 31 October 2018 (22). During this period of time 254 women had frozen their eggs for non-medical reasons, and 38 of them (15%) had returned to use their banked oocytes. The cumulative LBR/OPR was 63% for women 36–37 years of age at freezing and 26% for women 38–39 years of age at freezing. There were unfortunately no births or ongoing pregnancies for women who were 40 years of age or older at the time of the freezing. Although our study includes only a limited population, it clearly demonstrates that the chance of having a child drops with the age at freezing.

### Age-limits

In the work with national guidelines age-limits have been discussed. There may be reasons to consider age-limits both for freezing eggs and for using them. Since the probability of a pregnancy is highly related to the age of the woman at freezing, elective egg freezing is usually discouraged after the age of 38 years (18). It is, however, agreed that setting an upper age-limit for freezing is up to the individual clinic.

It is more difficult to define an upper age-limit for attempting pregnancy with frozen eggs. Pregnancies at advanced maternal age are generally considered more hazardous than at younger age. The risks of pregnancy diabetes, hypertensive disorders of pregnancy, and preeclampsia are increased, as is the risk of perinatal complications. The number of caesarean sections is also very high (28–31). Based on this knowledge and on international practice, the general recommendation in Sweden is not to thaw eggs at a higher age than 46 years. On the other hand, the surveys suggest that the risk of maternal and neonatal complications depend on the mother’s preconceptional health (28–31). SMER states that it is improper to define a certain age-limit as ageing is individual. According to SMER, the basis should be a woman’s medical capacity of coping with a pregnancy and the best interest of the future child, rather than her actual age (32).

### Social egg freezing

The expression ‘social egg freezing’ can be questioned since it implies that women deliberately postpone childbearing for various social reasons, e.g. career, studies, or simply because a child does not fit in to their present situation of life. Several studies have shown that this assumption is wrong. Most women who freeze eggs have a strong wish for a child, but wish to share their parenthood with a partner rather than being a single parent (33,34). Elective egg freezing is often a last resort. At Nordic IVF, where almost 300 women have banked their eggs, the mean age for freezing eggs is around 37 years, which is in line with others (20–22). It reflects the fact that women hope to find a partner and wait to the last second to freeze their eggs.

Even after thorough information, however, many women state that freezing is better that doing nothing, and that they would regret it if not (35,36). The women in the study by Gold et al. (35) also stated that they would have frozen earlier if they had been aware of the possibility.

Our mission as gynaecologists must be to inform that the possibility of having a child is greatest at younger ages, but also to inform about elective egg freezing alongside other reproductive alternatives. Egg freezing by choice means no guarantee to have a child but offers women a means to take control of their fertility. Many women are insufficiently aware that fertility drops with age, and, by adding information of the possibility to freeze eggs to the message of fertility decline, women may feel helped rather than lectured.
